# Tackling social determinants of dementia will require qualitative research with the public and professionals

**DOI:** 10.1002/alz.70767

**Published:** 2025-09-27

**Authors:** Timothy Daly

**Affiliations:** ^1^ Bioethics Program FLACSO Argentina Buenos Aires Argentina

1

In their scoping review, Walsh et al. found varying degrees of evidence for six classes of “social determinants of dementia” (SDOD), upstream features of the physical and social environment that produce, or protect against, dementia risk.^1^ The authors recognize that evidence is strongest for education and lacking for other SDOD, such as housing stability, and they understandably call for further causal research with rigorous methodologies.

Establishing causal evidence is one piece of the puzzle. However, the authors do not mention the need to integrate quantitative evidence with qualitative research with the public, policy makers, and health‐care professionals, which is equally necessary to make a public health approach to SDOD actionable and equitable. As an example, Dykxhoorn et al.^2^ conceptualized mental health priorities alongside members of the public with different levels of exposure to determinants, as well as policy makers and public health practitioners. For all of the known determinants, they asked the public and professionals to rank each determinant according to two criteria: its importance for lived experience, and its amenability to change.^2^ They then used this aggregated list to create a hierarchy of priorities for public mental health.

This synthesizing of different perspectives is important because the public, particularly those in situations of vulnerability, perceive “a clear hierarchy of influences” of factors influencing their health, which may be incorrectly “listed on par” in academic research.^3^ For instance, in a qualitative study with members of the public on how urban environments could support dementia risk reduction, Susanne Röhr et al.^4^ found that social inclusion and housing—which Walsh et al. found to have significant evidence gaps^1^—was the primary theme that emerged from interviews. This shows how academic research and lived experience can differ greatly. Moreover, in other qualitative studies, policy makers^5^ recognized the need to address SDOD, whereas general practitioners^6^ tended to stress individual behaviors, highlighting the need to work with different groups simultaneously to prioritize relevant, actionable fulcra for action against SDOD.

In summary, academic research, health policy making, and the lived experience of exposure to social determinants are very different realities. Just as Walsh et al. have brought together evidence to provide a state‐of‐the‐art review of the academic literature, further research and policy efforts should bring together different groups to establish priorities that address the needs of the public, professionals, and researchers by underlining the evidence, importance, and actionability of SDOD. Quantitative evidence alone cannot answer the question: What are the reasonable means required to reduce brain health inequities?^7^ To answer it will require integrating quantitative data from Walsh et al.’s approach with qualitative evidence, emphasizing the need for action while the evidence base is under construction.^8^

## CONFLICT OF INTEREST STATEMENT

Dr. Timothy Daly has no conflicts of interest to declare. All author disclosures are available in the supporting information.

## Supporting information



Supporting Information
